# Comparison of a Novel Liquid Embolic System with Commonly Used Embolic Agents in the Endovascular Treatment of Intracranial Dural Arteriovenous Fistulas: A Single-Center Experience

**DOI:** 10.3390/jcm13195899

**Published:** 2024-10-02

**Authors:** Zarko Nedeljkovic, Ivan Vukasinovic, Masa Petrovic, Aleksandra Nedeljkovic, Tijana Nastasovic, Vladimir Bascarevic, Mirko Micovic, Mihailo Milicevic, Marina Milic, Nemanja Jovanovic, Aleksandar Stanimirovic, Vuk Scepanovic, Danica Grujicic

**Affiliations:** 1 Clinic for Neurosurgery, University Clinical Center of Serbia, 11000 Belgrade, Serbia; 2 Center for Radiology, University Clinical Center of Serbia, 11000 Belgrade, Serbia; 3 Institute for Cardiovascular Diseases ”Dedinje”, Heroja Milana Tepica 1, 11000 Belgrade, Serbia; 4 Faculty of Medicine, University of Belgrade, 11000 Belgrade, Serbia; 5 Department of Anesthesiology and Resuscitation on Neurosurgery Clinic, University Clinical Center of Serbia, 11000 Belgrade, Serbia

**Keywords:** embolization, dural arteriovenous fistula, endovascular procedures, carotid cavernous fistula, liquid embolic agent

## Abstract

**Background/Objectives:** Endovascular embolization is an effective treatment option for cerebral arteriovenous malformation (AVM) and dural arteriovenous fistulas (DAVFs). The objective of this study was to assess the safety and efficacy of Menox^TM^ in patients with cranial dural arteriovenous fistulas. **Methods**: From January 2021 to January 2023, 19 patients with intracranial DAVFs underwent embolization procedures. All patients were treated by embolization with Menox^TM^ or/and in combination with other embolization products such as Onyx (Covidien, Irvine, California), PHIL (MicroVention, Tustin, California), and Squid (Balt Extrusion, Montmorency, France). Treatment approaches were selected depending on the anatomical location of the fistula. Patients were monitored and followed-up for 12 months. **Results**: The patients’ mean age was 56.26 ± 16.49 years. Of these 19 patients, 58% (*n* = 11) were treated with the Menox^TM^ liquid embolizing agent (LEA) alone or in combination with different LEAs, while *n* = 7 were treated with other LEAs and 1 patient was treated solely with coils. Complete occlusion of DAVFs with Menox^TM^ and other agents was evident in 68.4% (*n* = 13/19) of patients. Complete occlusion (100%) was observed in the sinus rectus, transverse sinus, and diploic veins of the orbital roof, while complete occlusion was observed in 50% of falcotentorial patients and 60% of superior sagittal sinus patients. The lowest rate of complete fistula obliteration was observed in the dural carotid cavernous fistula (CCF) group (25%). An intra-procedural adverse event occurred in one patient. No other post-procedural adverse events were noted. Furthermore, in patients treated with Menox^TM^, total occlusion was achieved in 72.7% (*n* = 8) of patients, whereas the non-Menox^TM^ group had 62.5% (*n* = 5) of patients with 100% occlusion and 37.5% (*n* = 3) of patients with subtotal occlusion. **Conclusions**: Outcomes using Menox^TM^ alone and in combination with other agents were effective, and it is safe for the treatment of dural arteriovenous fistulas.

## 1. Introduction

The etiology of dural arteriovenous fistulas (DAVFs) is still not fully understood. Several etiological factors have been hypothesized as possible causes of fistula formation, such as venous sinus thrombosis, previous surgical interventions, or trauma [1,2,3]. DAVFs, also referred to as arteriovenous malformations (AVMs), are abnormal shunts between the arterial and venous systems within the dura mater [4]. The clinical presentation of DAVFs varies depending on the location, and encompasses symptoms like headaches, pulsatile tinnitus, changes in the visual field, myelopathy, seizures, changed mental behavior, and cranial nerve palsies [5]. Approximately 20–33% of DAVFs are manifested through intracranial hemorrhage in initial stages [6].

Over the past two decades, the transarterial embolization technique has evolved as the predominant approach in the treatment of DAVFs [7]. Liquid embolic agents based on ethylene vinyl alcohol (EVOH) are mostly used for DAVFs, and therefore, understanding their properties is the essence of its successful treatment. Two treatment approaches are considered for DAVFs, transvenous and transarterial. However, in certain conditions a combined approach is inevitable. EVOH-based LEAs have several advantages over cyanoacrylate-based LEAs, such as a longer injection time, the possibility of rapid occlusion of the venous segment of the fistula, and usually requiring only one arterial feeder injection. Due to the existence of internal carotid artery and external carotid artery anastomosis, the transvenous approach remains the preferred approach in the case of CCF.

Despite significant advancements in the treatment of DAVFs over the years, the use of embolic agents continues to present several challenges. One of the main challenges in the treatment of DAVFs is achieving sustainable long-term occlusion, particularly in cases with complex venous drainage patterns. Current embolic agents, such as LEAs or coils, may not always provide complete occlusion, and thus may require multiple-stage treatment. Moreover, there can be a risk of recanalization or complications such as non-target embolization, leading to suboptimal patient outcomes. Therefore, the need for ongoing development of new embolic agents is paramount to offer better control, precision, and long-term effectiveness.

The introduction of new embolic agents to the market is reflective of the growing demand for materials to address current limitations. New agents are often designed to improve navigability, enhance penetration into smaller, distal feeders, or reduce the risk of complications. With regard to this, investigating new embolic materials is essential to optimize treatment outcomes, especially in cases for which existing options are inadequate. The goal is to achieve more effective and minimally invasive interventions for DAVFs, ultimately improving patient outcomes.

DAVF represents a wide range of symptoms, from subtle manifestations to a patient exhibiting severe neurological deficits in initial stages. Notably, DAVF leads to cerebral venous hypertension via retrograde drainage, mimicking symptoms associated with benign intracranial hypertension. Therefore, careful evaluation is of great importance in patients presenting with intracranial hypertension-like symptoms prior to lumbar puncture [8,9,10]. There are two widely used classification systems for grading the clinical behavior and the risk of intracranial hemorrhage in DAVF, the Cognard and Borden classifications, respectively [11]. The primary aim of our study was to assess the safety and efficacy of Menox^TM^ (Meril Life Sciences, Vapi, Gujarat, India) in the treatment of patients with cranial DAVF.

## 2. Materials and Methods

### 2.1. Study Design and Population

This was a single-center retrospective study that was conducted at the Department of Interventional Neuroradiology, Clinic for Neurosurgery at the University Clinical Center of Serbia. This study was performed in accordance with the International Code of Medical Ethics of the World Medical Association 1964 Declaration of Helsinki. Due to the nature of our study, it was exempt from obtaining prior approval by the Ethics committee. This study included 19 consecutive patients who were treated for intracranial DAVF and underwent an arterial, venous, or combined embolization procedure during the period from January 2021 to January 2023 at the Clinic for Neurosurgery at the University Clinical Center of Serbia. All patients were treated by embolization utilizing Menox™ liquid embolic agent or with other embolization products such as Onyx (Covidien, Irvine, CA, USA), PHIL (MicroVention, Tustin, CA, USA), Squid (Balt Extrusion, Montmorency, France), or detachable coils. Additionally, in some patients a combination of Menox™ and other embolization products were used; thus, a comparison was made based on whether or not Menox™ was used. Each treatment approach was selected depending on the operator’s preference, availability, and the anatomical location of the fistula. The use of an additional LEA other than Menox™ was determined upon the success of the progression of the LEA through the arterial system towards the proximal part of the drainage vein. Patients with spinal DAVF, direct carotid-cavernous fistula (Barrow classification type A), intracranial DAVF treated surgically, as well as patients without available clinical records and/or imaging studies and patients without a control clinical examination after procedural and radiological follow-up were excluded from this study.

### 2.2. Baseline Characteristics

Baseline data included age, sex, medical history (comorbidities), and initial presentation (bleeding or non-bleeding, symptoms, and pre-procedural modified Rankin Scale (mRS) score). Baseline characteristics of the study population are outlined in Table 1.

### 2.3. Procedural Characteristics

Procedural data included (1) the type of embolization approach (i.e., use of venous, arterial, or both routes); (2) embolization technique (liquid embolic agent or coils); (3) embolization result (complete or partial exclusion); (4) intra-procedural complications and adverse events after the procedure; (5) amount and time of injection of liquid embolic agent; (6) type of microcatheters used; (7) clinical follow-up taken after 6 months; and (8) imaging follow-up taken from the 9th to 12th months. Dural fistula grade was assessed through the Cognard DAVF scale [11].

Menox™ liquid embolic material is a non-adhesive liquid embolic agent consisting of EVOH polymer that allows multiple cycles of short-lived and continuous injections, dissolved in dimethyl sulfoxide (DMSO) and suspended in micronized tantalum powder to provide radiopacity. DMSO acts as a dissolving agent. Pure DMSO is injected to fill the dead space of the microcatheter prior to the injection of Menox™. Injecting DMSO prevents the unintentional precipitation of Menox™, which immediately begins when there is contact with water, saline solution, or blood. Menox™ advances into the vasculature with a lava-like flow pattern without any fragmentation during injection. Menox™ (LES) is available in three variants, the Menox™ Liquid Embolic System (18 (cSt) viscosity) (5% EVOH), the Menox 18™ Liquid Embolic System (20 cSt viscosity) (6% EVOH), and the Menox™ Liquid Embolic System (34 cSt viscosity) (7% EVOH). In our cohort, we used Menox 18™ exclusively.

## 3. Results

From January 2021 to January 2023, a total of 19 patients (male = 13 and female = 6) with cranial DAVF were treated. Among these 19 patients, a total of 23 procedures were performed. Of these 23 procedures, 4 were two-stage procedures. The mean age of all patients was 56.26 ± 16.49 years. Of the *n* = 19 patients, 58% (*n* = 11) were treated using Menox™ or a combination of Menox™ and another LEA, while the remaining patients were treated using another LEA without Menox™, except 1 patient, who was treated solely with coils. Of the 19 patients, *n* = 18 patients presented with symptoms; the three most common symptoms were headache, exophthalmos, and chemosis (Table 2).

Eight patients (42.1%) presented acutely with intracranial hemorrhage (five patients), seizure (three patients), and right-sided hemiparesis with motor dysphasia (two patients). Ten patients (52.6%) were symptomatic, and presented with paresthesia (one patient), tinnitus (three patients), progressive quadriparesis (one patient), headache (six patients), exophthalmos (four patients), and chemosis (five patients). One patient (5.3%) was asymptomatic, and four had a decline in visual acuity.

Furthermore, patients *n* = 10/19 presented with aggressive DAVF (type IIb or higher), *n* = 5/19 patients had type I or IIa DAVF, and *n* = 4/19 had CCF (Table 1). The major anatomical locations of DAVF were occipital (*n* = 5/19), CCF (*n* = 4/19), and tentorial (*n* = 3/19), and others were falcotentorial (2/19), temporal (2/19), transverse sinus (1/19), parietal (1/19), and orbital (1/19) (Table 3).

The majority of procedures were performed through the middle meningeal (*n* = 11/19), occipital (*n* = 3/19), and maxillaris arteries (*n* = 3/19) and the internal jugular vein (*n* = 2/19). All CCF procedures were performed through the inferior petrosal sinus. Commonly used microcatheters for injection were Apollo (*n* = 7/19; Covidien, Dublin, Ireland), Headway Duo (*n* = 5/19; MicroVention, Aliso Viejo, CA, USA), Sonic (*n* = 4/19; Balt), Headway 21 (*n* = 1/19; MicroVention), Headway 17 (*n* = 1/19; MicroVention), and Eclipse 2L (*n* = 1/19; Balt, Baltimore, MD, USA). Of 18 patients treated with LEAs, 11 were treated with Menox^TM^ alone or in combination with another LEA such as Onyx 18 (2/19), PHIL 30 (1/19), or Squid 12 (1/19), and/or detachable coils (3/19). The remaining seven patients were treated with another LEA such as Onyx (3/7), PHIL 25 (1/7), Squid 12 (2/7), or Squid 18 (1/7).

### 3.1. Post-Procedure Results

**Figure 1:**
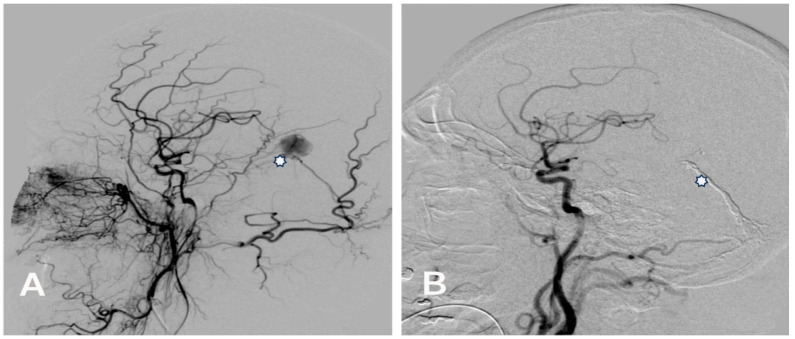
(**A**) Pre-embolization lateral angiogram of a Cognard type IV falcotentorial DAVF with venous ectasia (asterisk) presented with right-sided intracerebral hemorrhage treated with Menox 18 through the middle meningeal artery. (**B**) Control post-embolization angiogram of the right common carotid artery showing the complete occlusion of the DAVF and the cast of Menox 18™ (asterisk).

**Figure 2:**
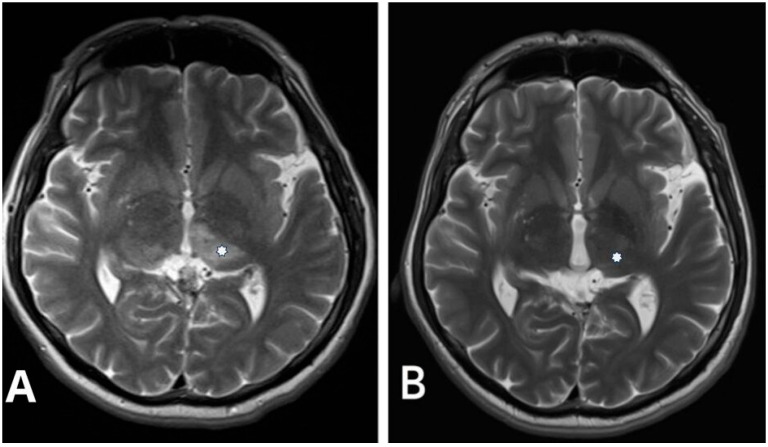
(**A**) Pre-procedural T2-weighted axial image in the same patient with Cognard type IV falcotentorial DAVF showing a bilateral thalamic edema (asterisk). (**B**) Control postprocedural T2-weighted axial image performed 2 years after intervention demonstrates complete resolution of the thalamic edema (asterisk).

Complete occlusion of the DAVF with Menox™ and other agents was evident in 68.4% (*n* = 13/19) of patients. Complete occlusion (100%) was observed in the sinus rectus, transverse sinus, and diploic veins of the orbital roof, while complete occlusion was noted in the falcotentorial sinus in 50% of patients (Figure 1 and Figure 2) and in the superior sagittal sinus in 60% of patients.

The lowest rate of complete fistula obliteration was observed for the dural CCF group (25%). An intra-procedural adverse event occurred in one patient. This involved a patient with a type IIa DAVF; the microcatheter ruptured during their procedure. No other post-procedural adverse events were noted. Furthermore, in patients treated using Menox™, immediate total occlusion was achieved in 72.7% (*n* = 8) of patients (Table 4).

### 3.2. Six-Month Follow-Up Results

Clinical outcome (mRS) follow-up visits were scheduled 6 months after endovascular treatment, and angiographic follow-ups were scheduled 9 to 12 months after embolization (Table 4). Forty-two percent (*n* = 8/19) of patients’ mRS scores were unchanged since their post-procedure visit, while 57.9% (*n* = 11/19) of patients had improved mRS scores. Angiographic controls showed 84.2% (*n* = 16/19) of patients with complete DAVF occlusion at the 9th to 12th month (Table 4).

## 4. Discussion

In the modern era, the management of DAVF has evolved significantly, with endovascular treatment emerging as the primary approach for many cases compared to alternatives such as microsurgical resection and radiosurgery [12,13]. This paradigm shift reflects the technological advancements and improved understanding of the underlying pathophysiology of DAVF. One notable advancement in DAVF treatment is the introduction of LEAs, which have added versatility to endovascular interventions. Currently, there are several LEAs available, each with unique properties and varying degrees of approval in different geographic regions. It is imperative to carefully consider the choice of LEA, considering its distinct characteristics, the angioarchitecture, and the individual patient’s condition [14]. In our study population, the arterial approach was the most frequently utilized (17/19 patients). However, only *n* = 7/17 achieved 100% occlusion immediately after the procedure. This result was comparable to the study by Hu et al., in which onyx achieved an 87% occlusion rate in *n* = 29/50 patients immediately after the procedure [15].

Symptomatology associated with DAVF can vary significantly, encompassing a spectrum from subtle manifestations to severe neurological deficits. In our study, most patients presented with a range of symptoms, with headache, chemosis, and exophthalmos being the most common. It is crucial to note that DAVF may lead to cerebral venous hypertension through retrograde drainage, sometimes mimicking the symptoms of benign intracranial hypertension. As such, careful evaluation and consideration of DAVF in patients with intracranial hypertension-like symptoms are warranted before performing lumbar punctures [8,9,10].

Hemorrhagic manifestations are one of the most critical issues in managing DAVFs due to the increased risk of rupture, particularly in higher-grade lesions. Therefore, classifying DAVF is essential for treatment planning and prognostication. Two commonly used classification systems, the Cognard and Borden classifications, are invaluable tools in this regard. In our study population, the Cognard classification system revealed a predominance of type IV DAVF (Figure 1 and Figure 2), characterized by retrograde leptomeningeal venous drainage with venous ectasia (Table 1). In patients with type IV DAVF, the most common symptoms were headache, nausea and vomiting, and neurologic conditions such as seizure and disorientation due to cerebral venous hypertension, which in our study was neuroradiologically manifested by bilateral thalamic edema in one patient (Figure 2). Classification helps tailor the treatment strategy to the specific characteristics of the lesion.

The choice of LEA also plays a pivotal role in achieving favorable treatment outcomes. In our comparative analysis, Onyx, Squid, and PHIL demonstrated similar embolic properties, despite structural differences. However, Menox™ exhibited distinct advantages. Notably, less Menox™ [2.0 ± 1.1 mL versus 1.2 ± 0.4 mL (*p* = 0.032)] was required to achieve comparable results compared to other LEAs, suggesting its efficiency and cost-effectiveness. However, larger studies, including a cost-effectiveness analysis, should be performed in the future to further explore our findings.

As with all LEAs, Menox™ also carries certain potential risks and limitations that warrant careful consideration. Specifically, in our study, we observed complete obliteration of the fistula in a significant proportion of patients (68.4%) following initial endovascular treatment, with only one reported complication related to microcatheter breakage. This can be possibly attributed to the viscosity and cohesive properties of Menox™, which may introduce technical challenges in certain anatomical locations. It is also essential to be aware of the risk of non-target embolization, especially in cases with intricate angioarchitecture. Further studies are needed to assess long-term outcomes and potential complications, as the introduction of any new agent requires a cautious approach to ensure patient safety.

Our results align with previous studies utilizing Onyx, in which high rates of fistula closure and relatively low complication rates were reported [10,16]. The study by Guedon et al. also demonstrated the efficacy of transvenous treatments for DAVF, further supporting the use of endovascular approaches [17]. The choice of the arterial approach, particularly through the middle meningeal artery, emerged as the most common technique in our study, enabling efficient treatment from a single arterial feeder. This approach minimizes procedural complexity while optimizing outcomes.

Overall, Menox™ presents a promising alternative in the armamentarium of LEAs, with the potential to enhance the efficacy of endovascular treatment of DAVFs. However, a synergistic approach that considers both its advantages and limitations relative to established agents like Onyx, Squid, and PHIL is crucial for optimizing outcomes and minimizing risks in clinical practice.

## 5. Conclusions

Our study contributes to the growing body of evidence supporting the efficacy and safety of endovascular treatments for DAVF, with a focus on the advantages of Menox™ as a novel LEA. The choice of approach and classification are critical factors in achieving successful outcomes in the management of DAVF. Our study demonstrated that it is suitable to use liquid embolic agents either independently or in combination, as they are effective and safe in both transarterial and transvenous approaches.

## Figures and Tables

**Table 1:** Study population characteristics.

	Menox™		
	No (*n* = 8)	Yes (*n* = 11)	*p*-Value
**Age**	59.4 ± 11.2	54.0 ± 19.7	0.50 ^a^
**Gender (male)**	6 (75.0%)	7 (63.6%)	0.50 ^b^
**Comorbidities (yes)**	6 (75.0%)	8 (72.7%)	0.60 ^b^
Hypertension	4 (50.0%)	6 (54.5%)	0.91 ^b^
Diabetes mellitus	2 (25.0%)	1 (9.1%)	0.85 ^b^
**Cognard Type**			
I	1 (12.5%)	1 (9.1%)	0.69 ^b^
IIa	2 (25.0%)	1 (9.1%)	
IIb	0 (0%)	2 (18.2%)	
III	1 (12.5%)	1 (9.1%)	
IV	4 (50.0%)	5 (45.5%)	
V	0 (0%)	0 (0%)	
**Bleeding**	1 (12.5%)	4 (36.4%)	0.24 ^b^
**Symptomatic**	7 (87.5%)	11 (100%)	0.23 ^b^
**Coiling**	1 (12.5%)	3 (27.3%)	0.44 ^b^
**Intraprocedural complications**	1 (12.5%)	0 (0%)	0.23 ^b^
**Adverse events after intervention**	2 (25.0%)	0 (0%)	0.08 ^b^
**LEA (mL)**	2.0 ± 1.1	1.2 ± 0.4	0.032 ^a*^

All values are shown as mean ± SD or *n* (%) depending on the type of variable. a = independent samples *t*-test, b = chi-squared. * *p*-value less than 0.05 was statistically significant.

**Table 2:** Patient presentation on admission.

Symptom	*n* (%)
Headache	6 (33.3%)
Chemosis	5 (27.8%)
Exophthalmos	4 (22.2%)
Decline in visual acuity	4 (22.2%)
Seizure	3 (16.7%)
Tinnitus	3 (16.7%)
Nausea	3 (16.7%)
Vomiting	3 (16.7%)
Ataxia	2 (11.1%)
Rightsided hemiparesis with motor dysphasia	2 (11.1%)
Motor dysphagia	2 (11.1%)
Bradypsychia	1 (5.6%)
Paresthesia of the left side of the head	1 (5.6%)
Facialis central facial palsy	1 (5.6%)
Multiple episodes of disorientation	1 (5.6%)
Inability to walk alone Quadriparesis	1 (5.6%) 1 (5.6%)

**Table 3:** Procedure characteristics.

Patient	Localization	Approach	LEA
1	Tentorial	Arterial	Onyx 18
2	CCF	Venous	Onyx 18
3	Parietal	Arterial	Squid 12
4	Sinus transversus	Combined	Onyx 18, Squid 12, Coils
5	Tentorial	Arterial	Menox™ 18
6	CCF	Venous	Menox™ 18, Coils
7	Tentorial	Arterial	Onyx 18
8	Falcotentorial	Arterial	Squid 12
9	CCF	Arterial	Menox™ 18
10	Occipital	Arterial	Menox™ 18
11	Occipital	Arterial	Menox™ 18
12	Orbital	Arterial	Menox™ 18, PHIL 30
13	CCF	Arterial	Menox™ 18
14	Falcotentorial	Arterial	Menox™ 18, Onyx18
15	Temporal	Arterial	Phil 25
16	Occipital	Arterial	Menox™ 18
17	Occipital	Arterial	Menox™ 18, Coils
18	Occipital	Arterial	Squid18
19	Temporal	Arterial	Menox™ 18, PHIL25, Coils

**Table 4:** Post-procedural results and mRS outcomes.

Patient	Immediate Post-Treatment Occlusion	mRS Before	6-Month mRS
1	100% occlusion	1	0
2	Subtotal occlusion	1	0
3	100% occlusion	3	1
4	100% occlusion	1	0
5	100% occlusion	2	2
6	100% occlusion	1	0
7	Subtotal occlusion	1	0
8	Subtotal occlusion	1	1
9	50% occlusion	1	1
10	100% occlusion	2	1
11	100% occlusion	3	3
12	100% occlusion	1	1
13	Suboptimal occlusion	1	1
14	100% occlusion	1	0
15	100% occlusion	1	0
16	20% occlusion	5	4
17	100% occlusion	4	4
18	100% occlusion	1	1
19	100% occlusion	4	2

The non-Menox™ group had 62.5% (*n* = 5) of patients with 100% occlusion and 37.5% (*n* = 3) of patients with subtotal occlusion. At the 9- and 12-month follow-ups, eight patients in the non-Menox™ group had 100% occlusion.

## Data Availability

Data presented in this study are available on request from the corresponding author due to privacy reasons.
